# Cases Treated in the Louisville Marine Hospital

**Published:** 1846-03

**Authors:** Charles L. Lyle, W. A. Harris

**Affiliations:** Louisville; Louisville


Art. II.—*Cases treated at the Louisville Marine Hospital.* **Reported by Mr. Charles L. Lyle, and Mr. W. A. Harris.**

*Hematemesis.*—James McGlinn, a native of Ireland, by occupation a ditcher, of intemperate habits, was admitted into the Hospital, August 11th, 1845. Three years ago he was engaged at work on the shores of the Tennessee river, and was compelled to labor constantly in water, sleeping in his wet clothes at night. After pursuing this life for several months he was attacked with intermittent fever. About this time he was advised to go to New Orleans for his health; he was improved by the travel and change of climate, but engaging in his laborious occupation in the suburbs of the City, in a short time he was taken with diarrhea, which persisted for several months before it was finally arrested—leaving him in a debilitated state, from which he never recovered. Six months ago, Hematemesis came on, after he had been enduring much fatigue and exposure to rain and cold, to overcome the effects of which he resorted to large potations of ardent spirits. The hemorrhage came on at night, after he had been indulging more excessively than usual in drink,—and recurred, at intervals, until he was admitted in August.

At this time his face was pale and blanched, his skin dry; pulse quick, small and feeble; tongue smooth and nearly exsanguineous. Nothing passed from his bowels but blood, which was thin, and of an arterial character. On the night of the 11th August, he vomited more than a quart of coagulated blood.

He was ordered to take acetate of lead, grs. iii., and opium, gr. i. The following day a fourth of a grain of tannin was exhibited every two hours. This course arrested the hematemesis as well as the discharge of blood by the bowels. He improved slowly under the administration of tinct. mur. fer. until about the first of September, when his extremities became œdematous, to which ascites followed shortly afterwards. The usual remedies for such cases were applied. The abdomen continued to enlarge, and became shining and tense.

Respiration hurried and difficult on account of the encroachment of the dropsy which compelled the patient to retain the semi-erect posture; pulse quick; a blowing sound of the heart.

An operation was deemed necessary for his relief, which was performed by Professor Gross on the 9th, by which upwards of two gallons of serum were removed.

10th. Saturated tinct. iodine was applied over the entire surface of the abdomen and a bandage applied. Was ordered tinct. fer. mur. and nutritious diet.

11th. Pulse 120, soft and small; skin dry and cool; tongue slightly furred and moist; bowels open.

12th. Treatment continued.

14th. Abdomen again becoming enlarged; fever and acceleration of the pulse during the evening.

15th. Water still increasing; prescription continued.

16th. Some fever, and at night tongue dry; bowels open; skin dry and hot; pulse still increasing in frequency.

17th. Abdomen painful from accumulation of water; some fluid escaping from the puncture; by means of cups a large quantity was drawn off, affording him much relief; large quantities continuing to pass through the puncture.

18th. Slept well during the night. Recurrence of hematemesis followed by syncope. Carb. ammonia, camphor mixture and brandy were exhibited for the purpose of stimulating him. To these sulphate of quinine, acetate of lead and tannin were subjoined, which constituted the treatment.

On the next day, about the same hour as on the preceding day, hematemesis again recurred, and was followed by fatal syncope; he died at 3 o'clock.

*Autopsy.*—On making an incision into the abdomen a large quantity of straw-colored serum was found; no attempt had been made at reparation of the puncture made by the trocar. Spleen enlarged, indurated, and its surface cartilaginous. The pleura also contained a quantity of straw-colored serum. The lungs pale, and containing two or three small calcareous deposits; otherwise healthy. Pericardium distended with serum; heart pale and flabby. The veins of the œsophagus, and of the cardiac extremity of the stomach, varicose. Stomach distended with dark blood. Several small ulcers communicating with the varicose veins in the œsophagus. The blood found in the stomach and intestines was not coagulated.

*Typhoid Pneumonia—Paralysis of the Leg from a Bed Sore.*—The subject of this case is a native of Germany, 22 years of age, of sanguineo-bilious temperament, a brewer by occupation, and of intemperate habits; he was admitted into the Hospital on the 21st of October last. He was attacked three weeks previous to his admission with a short, hacking cough, slight fever, and pain in the right side of the chest over the infra-mammary region; had been subject to repeated attacks of delirium tremens.

When admitted he manifested great muscular debility, and much nervous irritability, which resembled somewhat paralysis agitans, or, perhaps, more correctly the mercurial tremor, as it was an involuntary trembling of the voluntary muscles, when unsupported. When admitted his pulse was small, feeble, and quick; tongue coated with a dark brown fur, fissured, dry, and red at the tip and edges; teeth covered with sordes; countenance sallow, with occasional flushing of the cheeks; bowels much constipated; expectoration slight and pneumonic; tenderness over the epigastrium; pain on pressure over the cervico-dorsal region of the spine. Auscultation revealed the crepitus rhonchus over the anterior middle lobe of the right lung; slight broncophony and dulness on percussion over the lower lobe; respiration feeble over the inferior anterior portion of the left lung. Prominent among these symptoms were a want of muscular tonicity and exhaustion of nervous influence, produced by frequent attacks of mania a potu; the the almost invariable symptom of which, (tremors) became more prolonged after each successive attack, until the morbid condition was permanently impressed upon the brain and great nervous centres.

Calomel and Dover's powder were administered in small and repeated doses—three of the former and five of the latter—until slight ptyalism was induced. Dry cups and blisters were alternately applied over the seats of pain and tenderness; strict attention was paid to the secretions; this, together with stimulants and tonics, consisting, mostly, of camphor mixture, carb. ammonia, porter, and quinine, constituted the treatment from his admittance up to the first of November. At that time the crepitus rale could be heard over the entire region of the right lung; expectoration profuse; nervousness so increased that he could with difficulty partially protrude his tongue, which was smooth and red; great restlessness and extreme debility; perspiration sour and at times profuse. He continued in this state, without any manifest change, for nearly a week, when he fell into a state of coma, or stupor, which continued for several days. Brandy, carb. ammonia, and other powerful stimulants were given in large and frequently repeated doses, and counter-irritants were applied over the cervico-dorsal region of the spine. Under this treatment the delirium disappeared, and with it the severer pneumonic symptoms.

On the 15th November, his nates were found extensively inflamed and disposed to gangrene. The parts were protected by means of cotton as well as possible, but owing to his exhausted condition, and the long continued posture on his back, a deep and extensive slough was formed, embracing the whole of the sacro-iliac, and part of the lumbar region. A poultice made of ground flaxseed, charcoal, and cinchona, was applied, after having moistened the parts with a weak solution of nitric acid, which, with the internal tonic and stimulating treatment, rapidly promoted the detachment of the slough. The ulcer extended farther and more deeply on the right, than on the left side; and when the gangrenous parts were thrown off, it exposed a large part of the iliac bones. At the same time were manifested symptoms of irritation of the bladder, as pain on pressure over the hypogastric region and frequent micturition. These, however, gradually disappeared as the ulcer healed.

Dec. 1st. Pneumonic inflammation subsided; ulcer healthy and partially healed; countenance of the patient much bloated; considerable œdema of the lower extremities; dull pain frequently felt along the course of the gluteal, smaller sciatic and musculo-cutaneous nerves; accompanied by partial paralysis of the peroneal flexor and extensor muscles of the right leg, and toes, so that the foot is not only thrown out of its proper line of direction, but is also distorted and twisted upon itself, resembling somewhat that variety of club-foot termed *talipes varus.* Motion, or the slightest pressure over the lumbar region aggravates the pain, while the recumbent posture relieves it. There is also a loss of natural temperature in the affected leg and foot, which are constantly bedewed with a cold and clammy moisture. No involuntary retractions of the affected muscles can be excited, which is evidence that the process of ulceration affected not only the nerves of the part, but also the lower part of the spinal cord itself. For the relief of these symptoms the usual remedies have been resorted to, but as yet with but little success.

Feb. 1st. Ulcer has cicatrized; symptoms of incipient anasarca still continue; likewise tremors of the voluntary muscles, even when supported; pain along the course of the nerves of the leg and foot unabated; distortion of the foot somewhat augmented; secretions regular, and general health improved.

*Louisville, February*, 1846.

